# The utilization of virtual reality in the training of de-escalation of aggression for both providers and users of public and healthcare services from the new millennium to the COVID-19 era: a systematic review

**DOI:** 10.3389/fmed.2026.1657986

**Published:** 2026-02-05

**Authors:** Chao Tian Tang, Lucas Jun Hao Lim, Charlotte Tze Min Lee, Amanda Jia En Heah, Dexter Shih Tong Yeo, Shian Ming Tan

**Affiliations:** 1 Department of Psychiatry, Sengkang General Hospital, Singapore, Singapore; 2 Department of Mood and Anxiety, Institute of Mental Health, Singapore, Singapore; 3 MOH Holdings, Singapore, Singapore

**Keywords:** aggression, de-escalate, simulation training, violence, virtual reality

## Abstract

**Introduction:**

Virtual reality (VR) is a promising modality for the effective delivery of training in the de-escalation of aggression. This review aims to assess how VR has been utilized in training for the de-escalation of aggression among both providers and users of public and healthcare services from the new millennium to the COVID-19 era (2000–2022).

**Methods:**

A systematic review was conducted in accordance with a pre-registered protocol and adhered to the Preferred Reporting Items for Systematic Reviews and Meta-Analyses (PRISMA) guidelines. Seven key databases were searched, yielding 2373 studies for screening, of which 15 were included. Quality appraisal was performed using the widely used Critical Appraisal Skills Programme (CASP) tool.

**Results:**

VR training for the de-escalation of aggression was implemented using a variety of approaches, ranging from verbal interaction and emotion-recognition tasks to selection from multiple-choice response menus. Most studies assessed participants’ responses to the intervention, but none evaluated whether VR training had an impact at the organizational level. Overall, VR training content, modes of interaction, and reported improvements in participants’ confidence were viewed positively. However, some studies reported limitations related to the emotional impact, realism of virtual characters, and learning effectiveness. Additional features that may enhance the VR experience were discussed, with personalized, context-specific scenarios identified as an important area for development.

**Conclusion:**

Larger-scale studies are required to determine which specific training domains may benefit most from VR-based approaches, given the heterogeneity of populations and methodologies across studies conducted between 2000 and 2022.

**Systematic review registration:**

https://www.crd.york.ac.uk/PROSPERO/view/CRD42022307138, identifier CRD42022307138.

## 1 Introduction

The General Aggression Model proposes that aggression is largely based on the activation and application of aggression-related knowledge stored in our memory (1). These social knowledge structures develop over time via learning processes (1). Aggression can potentially be directed outward toward the environment, turned inward onto the self by a subject, or evaded in an attempt to minimize frustration (2). Notably, aggressive behavior toward staff in public healthcare services is a growing concern internationally (3–7). In a number of such studies, the majority of respondents reported various categories of aggression. Such a trend is alarming and demands an effective response, given the significant repercussions for staff, the public and healthcare systems as a whole (8–11). This is in addition to elevated levels of aggression which have been observed in various studies during the COVID-19 pandemic with significant implications for public and healthcare services (12–15). The frustration–aggression theory also posits that frustration which is commonly encountered in many public and healthcare services is a necessary and sufficient condition for aggression (16). Iterations of this theory have suggested that frustration foments aggression should the frustrated need pertain to significance and mattering which is relevant to essential services in the public and healthcare realm, introducing a fundamental social-psychological aspect to the dimensions of aggression and related frustration (17). The management of aggression is thus challenging given this context with different potential solutions viewed from the perspectives of providers who need to manage aggression and users or patients of public or healthcare systems whom this aggression can originate from (18).

Dimensions most negatively impacted by aggression include psychological ones, such as the development of depression and emotional consequences that affected work functioning (19). The relationships between these domains are complex and multi-directional (20). Exposure to psychological aggression at work negatively predicted performance at work (20). However, aggressive behavior itself may have different underlying motives and functions (21). Aggression in healthcare settings has also been associated with a higher incidence of burnout, lower patient safety, and more adverse events (11). A significant proportion of healthcare staff have also experienced high levels of stress as a result of such incidences (9). For example, it is estimated that workplace violence contributes to more than 17% of nurses leaving their jobs every year (22).

Sixty four de-escalation training evaluations conducted over a 40-year period, primarily in the fields of nursing and psychiatry showed that de-escalation training led to slight-to-moderate individual and organizational improvements (23). Aggression de-escalation training has been shown to have positive effects in inpatient psychiatric hospital settings internationally by reducing the incidence and severity of aggression, the use of physical restraint as well as unexpected events as a result of physical restraints (24, 25). Participants in such a de-escalation program also reported significantly increased feelings of safety regarding potential patient aggression (26). It also increased healthcare provider confidence to deal with aggression together with a positive change in attitudes toward workplace violence (27). However, the strongest impact of training appears to be on de-escalation-related knowledge, confidence to manage aggression and de-escalation performance based on a systematic review on mental health staff training (28).

Members of organizations can undergo some form of aggression de-escalation training. This type of training is often carried out in groups through role-play, where participants learn to communicate in a de-escalating manner toward the aggressive role-player. Such training has been shown to increase staff knowledge and confidence for such situations. Although this has been proven to be successful, it is costly, time-consuming and difficult to replicate (3, 29). The COVID-19 pandemic is also a sobering reminder that there is a need to allow for the training of providers of public and healthcare services remotely (30). A large systematic review showed that the majority of participants who participated in de-escalation training perceived that they had greater confidence and knowledge for handling confrontation situations, felt safer, and experienced less violence at work after training (31). Of the studies that measured incidents of physical violence, the majority of studies found a decrease in the number of violent incidents after the training; when violent incidences do occur, the majority noted reduced severity of violent incidents after the training (31).

When it comes to de-escalating aggression, the person being trained should be able to identify the emotional state of the aggressor to differentiate between proactive and reactive aggression and to react accordingly (32). Proactive aggression is driven by high levels of instrumentality and low emotionality to achieve a certain goal, whereas reactive aggression is an emotional reaction in response to provocation, frustration or a perceived threat (32). According to the type of aggression, the employee could communicate in a more dominant or empathic style (33). The training of healthcare staff is seen as a key element to the prevention and management of violence and aggression but questions remain as to the effectiveness of these programs in preparing staff to apply this to clinical practice (34). A review of current training systems have recommended that training should be on real life problems, be engaging, integrate decision-making, planning, organization, skill building and cover a range of interventions which can be difficult to achieve (34). Virtual reality (VR), on the other hand, serves as a promising tool to train users to master these skills required for effective aggression de-escalation. This is backed by encouraging results of initiatives that uses simulated environments with virtual humans for communication and social skills training (35–38). VR provides a fully immersive and interactive virtual world that can be utilized to present real world scenarios and invoke relevant emotions in the participants. Another thematic synthesis highlighted that the emphasis on de-escalation techniques is reactive, rather than preventive of, patient violence (39). Hence, de-escalation of aggression cannot be considered alone without the aspect of training end-users.

A review on the practical use of immersive VR in forensic psychiatry and relevant adjacent psychiatric and forensic fields from 2016 to 2020 showed that there is a lack of large randomized controlled trials of current assessments or treatments using VR simulation in forensic psychiatry showing clear advantages of VR over standard practice (40). In addition, recent published papers of VR in aggression de-escalation have discussed the needs of potential end-users, explained the algorithms of its computational models, and performed pilot studies to assess the results of their VR intervention (41–43). Creating and implementing a VR program can be potentially costly, time-consuming and requires in-depth technical knowledge (44). Hence, it is necessary to understand the advantages of current VR interventions in this field alongside their limitations and key features in order to refine future VR programs. Given the increasing complexities and diversities of healthcare systems with a mix of public-private partnerships displayed in recent typologies, it is essential to ensure that information from different perspectives of the public and healthcare spectrum is captured and included to better reflect the use of VR in this area (45, 46). Given the varied range of interventions with various different target populations and outcome measures both quantitatively and qualitatively, we aim to perform a systematic review that provides an overview on VR interventions in the field of aggression de-escalation training for both providers and users of public and healthcare services to address this gap in the literature.

## 2 Materials and methods

A systematic review was chosen as it allows us to synthesize the available published evidence base in this emerging area of interest. Our methodology was developed in accordance to The Preferred Reporting Items for Systematic Reviews and Meta-Analysis (PRISMA) guidelines. Studies were included if they fulfilled criteria based on the PICO (population, intervention, comparison and outcome) model (47), displayed in Table 1. Given the novelty of this intervention, we opted to include conference proceedings to capture emerging trends regarding this promising modality of training.

**TABLE 1:** Criterion for selection of studies.

Criterion	Criterion description
Participants	Providers and users of public and healthcare services
Interventions	Description of interventions involving VR in the training of de-escalation of aggression
Comparative group	Studies with and without comparative groups, not limited to non-virtual reality modalities in the training of de-escalation of aggression
Outcome measures	Outcome measures: 1. changes in aggression post-intervention without limitations on specific measurement tools 2. other changes in related measures such as emotional, social or cognitive outcomes 3. clinician or user satisfaction levels 4. degree of learning through the above modalities
Study type	Peer-reviewed papers, conference proceedings
Language	English
Date of Publication	Year 2000 onwards
Exclusion	Studies not published in the English Language

On 30th January 2022, 7 key databases [Pubmed, Scopus, Web of Science (WOS), IEEE Xplore, Education Resources Information Center (ERIC), PsycInfo, and the Cumulative Index to Nursing and Allied Health Literature (CINAHL)] were searched. The exact search strategy is detailed in Table 2.

**TABLE 2:** Search strategy.

Database	Results	Keyword search
Pubmed	398	(“virtual real*” OR “virtual environ*” OR “virtual experience*” OR “serious gami*” OR “serious game*”) AND (“aggress*” OR “violen*” OR “crisis intervention” OR “agitat*” OR “agonist*” OR “danger*” OR “threaten*” OR “combat*” OR “disrupt*” OR “angry” OR “anger”) AND (“educat*” OR “learn*” OR “teach*” OR “train*” OR “intervent*” OR “de-escalat*” OR “de escalat*”)
Scopus	1657	(TITLE-ABS-KEY(“virtual real*” OR “virtual environ*” OR “virtual experience*” OR “serious gami*” OR “serious game*”)) AND (TITLE-ABS-KEY(“aggress*” OR “violen*” OR “crisis intervention” OR “agitat*” OR “agonist*” OR “danger*” OR “threaten*” OR “combat*” OR “disrupt*” OR “angry” OR “anger”)) AND (TITLE-ABS-KEY(“educat*” OR “learn*” OR “teach*” OR “train*” OR “intervent*” OR “de-escalat*” OR “de escalat*”))
WOS	1019	(TS = (“virtual real*” OR “virtual environ*” OR “virtual experience*” OR “serious gami*” OR “serious game*”)) AND(TS = (“aggress*” OR “violen*” OR “crisis intervention” OR “agitat*” OR “agonist*” OR “danger*” OR “threaten*” OR “combat*” OR “disrupt*” OR “angry” OR “anger”)) AND (TS = (“educat*” OR “learn*” OR “teach*” OR “train*” OR “intervent*” OR “de-escalat*” OR “de escalat*”))
IEEE Xplore	185	(“virtual real*” OR “virtual environment” OR “virtual experience” OR “serious gaming” OR “serious game” OR “serious games”) AND (aggress* OR violent OR violence OR “crisis intervention” OR agitated OR agitation” OR agonist OR dangerous” OR threaten OR combat OR disrupt OR angry OR anger) AND (education OR educational OR learn OR learning OR teach OR teaching OR train* OR intervention OR “de-escalat*” OR “de escalat*”)
ERIC	70	(“virtual real*” OR “virtual environ*” OR “virtual experience*” OR “serious gami*” OR “serious game*”) AND (“aggress*” OR “violen” OR “crisis intervention” OR “agitat*” OR “agonist*” OR “danger*” OR “threaten*” OR “combat*” OR “disrupt*” OR “angry” OR “anger”) AND (“educat*” OR “learn*” OR “teach*” OR “train*” OR “intervent*” OR “de-escalat*” OR “de escalat*”)
PsycInfo	389	(“virtual real*” OR “virtual environ*” OR “virtual experience*” OR “serious gami*” OR “serious game*”) AND (“aggress*” OR “violen*” OR “crisis intervention” OR “agitat*” OR “agonist*” OR “danger*” OR “threaten*” OR “combat*” OR “disrupt*” OR “angry” OR “anger”) AND (“educat*” OR “learn*” OR “teach*” OR “train*” OR “intervent*” OR “de-escalat*” OR “de escalat*”)
CINAHL	160	(“virtual real*” OR “virtual environ*” OR “virtual experience*” OR “serious gami*” OR “serious game*”) AND (“aggress*” OR “violen*” OR “crisis intervention” OR “agitat*” OR “agonist*” OR “danger*” OR “threaten*” OR “combat*” OR “disrupt*” OR “angry” OR “anger”) AND (“educat*” OR “learn*” OR “teach*” OR “train*” OR “intervent*” OR “de-escalat*” OR “de escalat*”)

The study protocol was published in The International Prospective Register of Systematic Reviews (PROSPERO) (reference number CRD42022307138).

**FIGURE 1:**
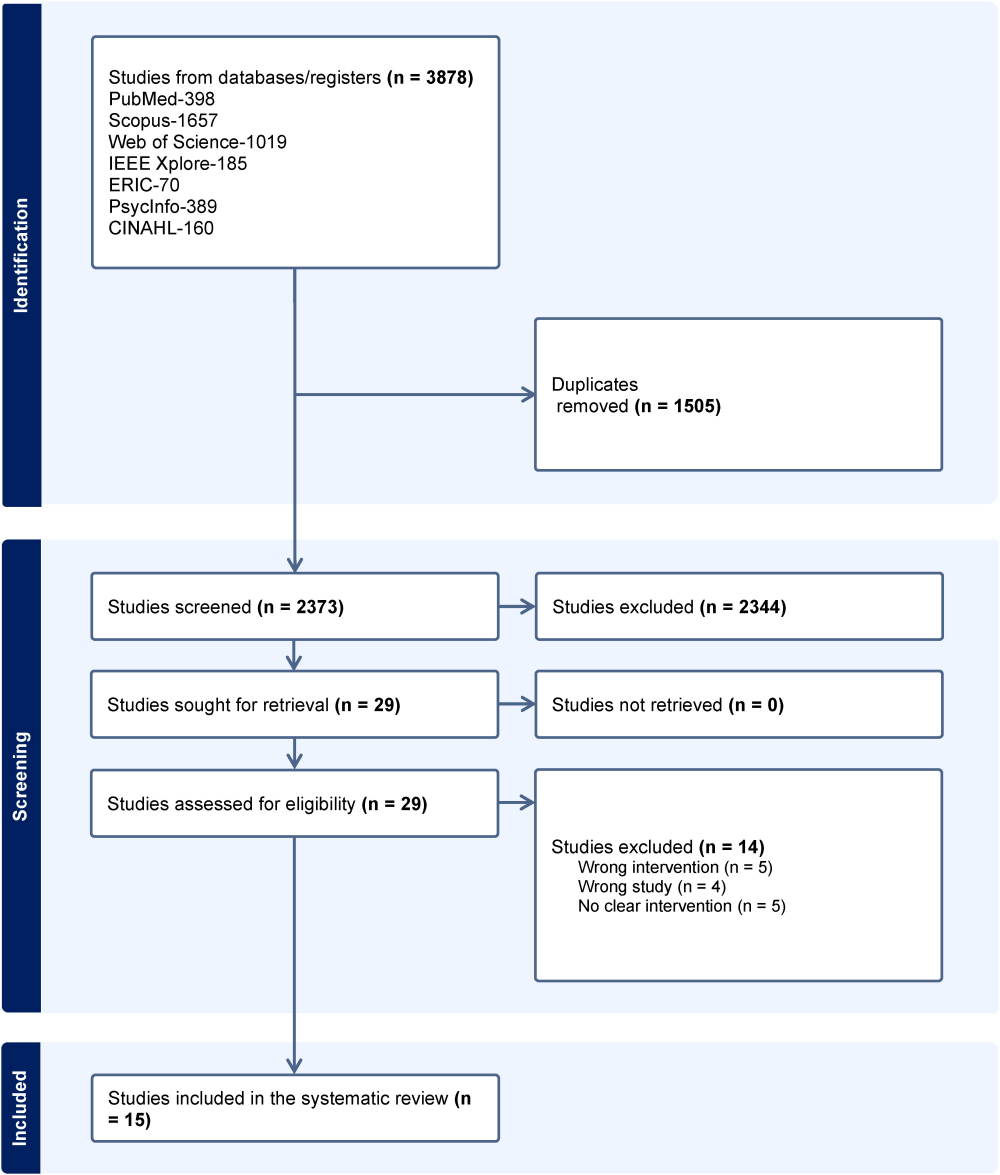
PRISMA flow diagram.

There were no restrictions on the type of study, but there must have been a clear description of programs involving VR in the training of de-escalation of aggression by both providers and users of public and healthcare services. Utilizing Covidence software, amongst the 3878 studies found, 1505 were duplicates. Two independent reviewers screened 2373 study titles and abstracts against the inclusion and exclusion criteria followed by full-text review of the selected studies to determine their eligibility. Any discrepancies between the two authors were resolved by discussion before acceptance for analysis. A third author was brought in when there were unresolved discrepancies. Inter-rater agreement was not obtained, however, as was obtained iteratively through discussion at each stage rather than recorded quantitatively within the screening software. Fifteen studies were obtained from this process. The PRISMA flow diagram is illustrated in Figure 1.

Study details including publication details, study design, aims, sample population, details of the VR program or intervention utilized in the training of de-escalation of aggression, study instruments and study outcomes were extracted using a data extraction form. One author independently extracted data from each paper which were then verified by a second author. Any discrepancies identified during verification were resolved through discussion, with referral to a third reviewer when consensus could not be reached.

Quality assessment of the studies was performed using the Critical Appraisal Skills Program (CASP) tool (48). The Critical Appraisal Skills Program (CASP) tool is one of the commonly used tools for quality appraisal in both in health-related qualitative evidence syntheses and quantitative studies, with endorsement from the Cochrane Qualitative and Implementation Methods Group (49, 50). Design-appropriate CASP checklists were used for different study types: the CASP Randomized Controlled Trial checklist for RCTs, the CASP Cohort Study checklist for cohort studies, and the CASP Qualitative checklist for qualitative studies. Descriptive studies were not assessed however given the lack of specific CASP tools. Kirkpatrick’s four-level evaluation model was used as a framework for categorizing the evaluation approaches used in the studies (51). This model is one of the most widely used models in assessing training outcomes and has been used in a number of evaluations for virtual training programs (52–54). This four-level evaluation model starts with Level 1 describing how the participants reacted to the educational program or training. Level 2 of this model assesses the extent to which the participants have learned from the program and Level 3 examines whether the participants are utilizing their new knowledge. Level 4 assesses if the program has a positive impact on the whole organization. Two authors independently assessed the studies for the quality assessment and Kirkpatrick’s levels of evaluation; inconsistencies were resolved through consultation with a third author.

We note that review spans many populations and settings where the following operational definitions and taxonomy may assist readers further.

1. Aggression: Includes reactive versus proactive forms, patient-initiated versus staff-initiated aggression, and verbal versus physical manifestations, as reported by the primary studies.
2. De-escalation training targets: Includes verbal and communication strategies, emotion recognition and perspective-taking, decision-making under stress, self-regulation, and team-based communication skills.
3. VR modality: Includes differentiating between fully immersive head-mounted display systems and non-immersive desktop simulations, as well as key design features such as avatar interactivity, branching dialogue versus fixed or multiple-choice interactions, adaptive difficulty, and the use (or absence) of physiological or performance-based feedback.

## 3 Results

The 15 studies included describe various interventions involving VR in the training of de-escalation of aggression. Tables 3–5 summarize the main elements of each study.

**TABLE 3:** Quantitative studies.

Author (country)	Year	Study design	Population	Sample size	Summary of the relevant aims	Intervention (majority of studies without a comparison)	Summary of the relevant results	Kirkpatrick level	CASP score
Smeijers et al. (41) (Netherlands)	2021	Randomized controlled study	Forensic psychiatry patients with aggression regulation problems	30	Assess changes in aggressive behavior and anger after using the Virtual Reality Game for Aggressive Impulse Management (VR-GAIME)	Patients experienced VR scenarios where they encountered avatars which allowed them to hone their emotion recognition skills, in addition to conventional aggression replacement training.	No difference was found between VR-GAIME and the control game in the improvement of aggressive behavior, but VR participants reported benefits in self-reflection and gained insight into their own behavior or triggers.	1, 2, 3	22
Tuente et al. (42) (Netherlands)	2020	Randomized controlled study	Forensic psychiatry patients with aggressive behavior	128	To assess if Virtual Reality Aggression Prevention Therapy (VRAPT) decreases aggressive behavior	VR exercises include learning skills such as facial emotion recognition, de-escalating aggressive behavior in others, and practicing these skills in interactive virtual role play.	VR participants did not have significant decreases in aggressive behavior. However, anger control and impulsiveness improved significantly in the VR participants group.	1, 2, 3	16
Alsem et al. (43) (Netherlands)	2021	Cohort study	Children with aggressive behavior problems	6	To assess the feasibility and effectiveness of interactive VR in decreasing aggressive behavior in children	The children undergo VR anger-provoking scenarios where they can practice emotional regulation and social information processing to decrease their aggressive behavior.	Therapists reported treatment feasibility and parents reported a decrease in children’s aggression over the treatment period.	1, 2	18
Halem et al. (55) (Netherlands)	2019	Cohort study	Mental healthcare professionals	31	To investigate the acceptability of VR aggression de-escalation training and potential impact on confidence in coping with aggression	VR role play with an aggressive avatar controlled by a trainer/actor.	Confidence in coping with patient aggression increased directly after the VR role play, especially in professionals less confronted with aggression.	1, 2	15
Ryu et al. (56) (Republic of Korea)	2016	Cohort study	Patients with Destructive and Impulse-Control Disorders	7	To assess the effectiveness of immersive Anger Management Virtual Reality Cognitive Behavior Therapy Program (AMVR-CBTP) to treat anger management and Destructive and Impulse-Control Disorders (DICD)	VR anger-provoking scenarios where patients could practice de-escalation and CBT techniques.	AMVR-CBTP intervention was effective in restoring abnormal brain activation waves and increasing associated anger control in individuals with DICD.	1, 2, 3	17
Bosse et al. (3) (Netherlands)	2016	Cohort study	Public service workers (public transport and police academy participants)	24	Use of VR for public service workers to practice aggression de-escalation skills, train social and communicative skills	Trainee interacts with aggressive virtual character in VR simulated environment and chooses appropriate response. Difficulty level of scenario changes based on trainee’s performance and physiological state.	Participants were positive about the content of scenarios and interaction possibilities, but were less convinced about the emotional impact of the VR scenarios.	1	14
Bosse et al. (57) (Netherlands)	2015	Cohort study	Professional train conductors and tram drivers	24	To assess the efficacy of VR training on improving aggression de-escalation competency of public service workers	Trainee interacts with aggressive virtual character in a VR simulated environment and chooses the appropriate response. Difficulty level of scenario changes based on trainee’s performance.	Participants had increased understanding of aggression de-escalation but were not able to translate this to correct decisions on actions to take in a given scenario.	1, 2	16
Bosse et al. (33) (Netherlands)	2015	Cohort study	Students or teachers at a high school	30	To explore the impact of threatening virtual stimuli on performance during emotion recognition tasks	Participants performed 2 tasks, an emotion recognition task and a mathematical task, each under 2 different conditions – a normal circumstance and a “stress” condition with threatening stimuli.	There was a negative impact of threatening stimuli on the emotion recognition task, but not on the mathematical task.	1	18

**TABLE 4:** Qualitative studies.

Author	Year	Study design	Population	Sample size	Summary of the relevant aims	Intervention	Summary of the relevant results	Kirkpatrick level	CASP score
Garcia et al. (58) (United States)	2021	Exploratory study	University students	4	To obtain feedback on the usefulness of a VR bystander de-escalation application involving bystander interventions in scenarios with aggression and conflict	A VR bystander de-escalation application was introduced to participants involving bystander interventional scenarios with aggression and conflict.	The intervention can change the perceptions of participants who have not been through the scenarios in real life. Participants also felt that VR was useful to prepare themselves mentally and emotionally for a similar situation.	1	16
Kim et al. (59) (Republic of Korea)	2021	Descriptive	Caregivers of dementia patients	77	To evaluate the effectiveness of a VR program for caregivers to enhance the practical competency of caregivers caring for dementia patients with behavioral and psychological problems of dementia including aggression and delusions	Caregivers experienced a VR case of male dementia patient who refused bathing help with aggressive behavior.	Participants reported an increased confidence in caring for dementia patients and felt that VR simulation training would be useful before starting caregiver work.	1, 2	16
Blakendaal et al. (60) (Netherlands)	2018	Focus group	Researchers and public transport domain experts	9	Focus group on the trial demonstration of a VR application for training of aggression de-escalation	VR scenario where participant converses with an aggressive virtual agent, identifies the type of aggression, choose the correct communication style and decides on reporting the incident.	Participants were positive about the training content and mode of interaction, but were less satisfied with the emotional impact and engagement during VR scenarios.	1	11
Blakendaal et al. (61) (Netherlands)	2018	Exploratory study	Academic students aged 18 years and above	12	To explore the usefulness of biofeedback in VR aggression de-escalation training	Adaptive VR scenario where the participant converses with an aggressive virtual agent and the biofeedback display is used to show the participant their stress level.	Participants reported a preference of seeing the biofeedback display over no display with an impact on stress and arousal.	1	14

**TABLE 5:** Descriptive studies.

Author	Year	Study design	Summary of the aims of the study	Description of interventions
Bosse et al. (62) (Netherlands)	2014	Descriptive	To develop a VR training system for aggression de-escalation that can be adapted to a participant’s performance for public service workers	Participant interacts with an aggressive virtual character in a VR simulated environment, identifies the type of aggression and chooses an appropriate response. Difficulty level of scenario changes based on the participant’s performance.
Bosse et al. (63) (Netherlands)	2014	Descriptive	Presents a computational model of interpersonal aggression used for VR where trainees can interact with aggressive virtual characters	Trainee responds to events in VR scenario involving aggression. Trainee’s behavior will be monitored by software and personalized support will be given.
Liu et al. (64) (United States)	2019	Descriptive	Creating a VR environment to aid children in anger de-escalation and management	Users enter a passive calming VR environment for de-escalation, then a CBT environment for active anger management training.

For the 6 cohort studies, the mean CASP was 16.4 (range from 14 to 18, standard deviation of 1.64) out a maximum of 24 points. The two RCTs scored 16 and 22 out of a maximum of 26 points. For the 4 qualitative studies, the mean CASP score was 14.4 (range from 11 to 16, standard deviation of 2.06) out of a maximum of 20 points. Further details are in the Supplementary material. There were no pre-specified scoring rules where there is no universally accepted cut off scores to classify summative scores from the CASP but extrapolating from studies which have done so, the quality of the studies would be considered to be at least of medium quality (65, 66). Using Kirkpatrick’s model of evaluation, all the remaining 12 studies assessed for participants’ reaction to the VR intervention (level 1). Six of these assessed the degree of learning (level 2) while only three studies attempted to determine whether there was any change in participant behavior (level 3). There were no studies that assessed if the VR intervention impacted an organization as a whole (level 4).

The Level 1 studies showed that participants generally had positive views about the VR training content and mode of interaction. However, they expressed reservations regarding the emotional impact and engagement in the virtual situation compared to real life scenarios due to perceived inadequate realism of the virtual characters (3, 57, 60). Despite this, VR training was reported to increase confidence in coping with aggressive incidents among mental healthcare professionals as well as caregivers of dementia patients (55, 59). VR training also seemed to be particularly useful for those with no prior experience with aggressive scenarios in real life (55, 58). User satisfaction was also seen in groups that were often more difficult to motivate or more resistant to therapy (42). Therapists of children with aggressive behavior also expressed high appreciation for the VR treatment session, expressing satisfaction with treatment feasibility and the delivery of content (43).

A few studies explored additional features that may enhance the VR experience and hence effectiveness. One study showed that the use of a biofeedback display during the VR session increases the user’s self-awareness of their emotional state and stress level (61). This can be useful in training users to manage their stress level and emotional state using relaxation techniques or otherwise. The ability of VR training systems to monitor users’ behavior and performance also allowed for a more personalized training experience. A few studies incorporated adaptation mechanisms, where the VR training system adjusted the difficulty level of the scenario based on the user’s performance, sustaining user engagement and motivation (3, 57, 62). VR programs could also monitor users’ behavior and physiological state, to generate personalized support and feedback (3, 57).

## 4 Discussion

Our systematic review reports on the role that VR has in aggression de-escalation in various settings based on existing literature. The 15 papers have a diverse group of participants and outcomes with different training programs where the population studied ranged from both patients to healthcare staff. Given the heterogeneity, it is hard to compare directly between the various VR training programs. There were concerns with a lack of a realistic “emotional threat” when faced with virtual aggressors where users did not feel as intrinsically threatened by the virtual aggressor as they would have been in real life (33). This suggests that more realistic and immersive encounters with virtual aggressors may be necessary, which may be achieved by improving both the hardware and the software of VR. “Presence” is considered as the propensity of people to respond to virtually generated sensory data as if they were real (67). It has been shown to be a key factor in VR exposure therapy for acrophobia and anxiety where it may be a necessary but insufficient requirement for a successful VR exposure program (68, 69). However, this was not a focus in the studies we reviewed where the lack of a focus on presence in the studies may have also contributed to some of the negative outcomes. Future trials will also benefit from any presence metrics and linking them to learning outcomes.

The use of motion sensors, 3D VR headsets as well as tracking of physiological stress levels and run-time biofeedback displayed on the VR screens can enhance immersion, emotional threat and arousal in VR users (60, 61). These findings by another study highlight that aggression de-escalation training programs had a greater impact on knowledge and confidence in handling aggressive patients, as compared to actual reductions in violent and aggressive incidents (70). Hence, simulating the emotional impact and threat that is experienced in real situations is also pivotal for users to practice to translate their theoretical knowledge of aggression de-escalation practically. However, biofeedback has limitations including complex mechanisms in relation to the expected response for example when looking at slow-paced breathing on heart rate variability (71). It is also difficult to prove that that the training was causally related to the physiological changes during ensuing the stress experience (71).

The results also suggest that for VR to effectively deliver teaching content and keep users engaged, it needs to continuously stimulate users as they progress in competence while moderating the difficulty level to their current level of competence. This has been shown to sustain the engagement and motivation of users in clearing and progressing in difficulty levels (41). Another method to ensure adequate adaptation would be to provide personalized feedback incorporating scenario-based feedback on the user approach to de-escalating aggression and the correlation to their physiological stress levels and emotional states (72). The difficulty of computational models of virtual aggressors should also be adjusted based on user feedback, and adapted to user’s competence to avoid unnecessary discouragement and loss of interest (61). This is similar to a review done on forensic psychiatry patients where the interventions in which a trained therapist controlled avatars in real-time appear to be more individualized than the more automated VR interventions requiring little provider input (40).

Our study has several strengths. Quality assessment of studies indicates that the studies scored were generally of robust quality where there are no clear guidelines regarding categorization of the studies although grading strategies by author consensus have been deployed by existing published peer-reviewed systematic reviews (65, 66, 73). A wide number of databases were also searched to reflect the interdisciplinary nature of this topic of study to reduce publication and selection bias. Conference papers were also included to allow the latest developments to be captured with quality assessment done as well. This inclusion of conference papers was also to increase the comprehensiveness and precision of the review and to decrease the potential impact of publication bias (74–76).

Our study’s limitation includes that there is the potential for a language bias given that we only searched for papers in the English language. There was a disparity in the number of papers from each database as well which may have been due to indexing differences. The limitations of these studies include the relatively smaller number of participants in the studies. This is due to the novelty of VR in aggression de-escalation practice, where some of these papers were more exploratory in nature. Given that this was the case, it was expected that outcome measures were not very clearly defined in a number of studies in particular pilot studies. There was also significant heterogeneity between the studies as there was a varied approach to conceptualizing and executing VR in aggression de-escalation given the different target populations in different settings hence it was not possible to aggregate the studies quantitatively. For example, Smeijers et al performed a randomized controlled study looking at changes in aggressive behavior via VR scenarios for forensic patients whereas Bosse et al. performed a cohort study on professional train conductors and tram drivers to see if it improved aggression de-escalation competency (41, 57). We included studies from the year 2000 onwards where VR technology has seen significant change over the years. From the new millennium, information technology experienced another phase of development where new technologies made it possible to project a VR environment and to simulate the consequences in advance (77). Due to the decentralization of processing capacity and the subsequent development of specific applications to support individualized decision-making, a new relationship with technology has been generated where the relationship is no longer subject to the time constraints of centralized systems (77). We are also mindful of potential response bias due to social desirability from self-reported questionnaires in the studies we examined (39). In addition, there may have been reporting biases where studies may have selectively reported favorable outcomes on VR. Inter-rater reliability was also not assessed between the assessors.

As the search encompassed the year 2000 to 2022. There is the risk that the conclusions no longer reflect the current evidence base, especially in a rapidly developing field such as this. In addition, our use of the CASP does not provide domain-specific bias judgments specific bias domains comparable to tools such the Risk of Bias 2 (RoB 2) and Risk Of Bias in Non-randomized Studies - of Interventions (ROBINS-I) where our appraisal should therefore be interpreted as an assessment of methodological rigor and reporting quality rather than a formal classification of risk of bias. The systematic review also contains technology-era bias, given the range of years the studies were published in 2000–2022. In this timeframe, a number of different versions of VR devices and software would have been used which could make it difficult to compare studies when it comes to issues such as feasibility and effectiveness. Heterogeneity was also detected when it came to the outcome measures, and how “aggression reduction” was operationalized in particular. Self-reported confidence or acceptability of a de-escalation intervention was used as a proxy in many of the studies instead of more objective or behavioral measures of de-escalation performance, which may make the studies more difficult to compare and may weaken the conclusions about transfer of skills to daily practice. Finally, problems were also identified when it came to the transfer of skills to daily practice and the fidelity of the VR simulation. Some studies had reported concerns about that the realism and emotion impact of avatars and scenarios. This may be problematic because the emotional threat and human relationships are key elements of a de-escalation procedure, and hence insufficient realism of virtual humans may be a barrier for transfer of skills from a virtual world to a real world.

While this review article was limited to articles from 2000 to 2022, we also found some additional studies beyond this timeframe that involved emergency responder and paramedic-relevant VR studies which is an acute, high-stakes setting. One study in 2025 was conducted with 37 paramedic students who underwent either VR-based training or conventional training using mannequins and real-world equipment where the VR group demonstrated significantly lower mental demand and frustration levels compared to conventional training (78). Another in the same year looked at performance indicators based on visual attention, triage performance, and information transmission using immersive VR where it was concluded that immersive VR based mass casualty incident scenarios proved to be a valuable tool for assessing the performance of medical first responders (79). Another systematic review in 2023 which looked at VR in simulation-based emergency skills training found that there may be educational benefit to using VR in the context of simulation-based emergency skills training including knowledge gain and retention, skill performance, acceptability, usability, and validity (80). These systems delivered education in a variety of areas including emergency medicine, equipment training, obstetrics, and basic/advanced life support where the subjective potential advantages of this technology included realism and time-effectiveness (80). Reports of adverse events were also low in frequency across the included studies which increases the ability of this to be generalized to clinical practice (80).

Our paper highlights the overall progress of VR intervention for aggression de-escalation, the conditions necessary for further enhancement of VR intervention and settings where VR interventions are most beneficial which can assist policymakers and researchers in specific domains of interest. The findings of our paper shows that VR also holds promise in the field of public service. Public service workers (such as police, public transport and public healthcare) can benefit from aggression de-escalation training due to the wide arrays of scenarios in the public service settings which can arise to conflicts (3). Another study has highlighted the lack of tracing and testing of innovations in de-escalation training in the de-escalation of aggressive situations during police training where our study provides specific scenarios where such interventions can be potentially useful (23). Key important features such as personalized feedback and individualized adaptation of difficulty levels to user competence can aid VR developers and subsequently policymakers or healthcare institutions who intend to utilize VR intervention for the training of de-escalation of aggression in prioritizing specific areas of development. This is especially important given the various other competing modalities of training with budgetary demands on other areas of an institution.

## 5 Conclusion

It is difficult for us to have a general conclusion given the heterogenous population and methodologies in our study. Conditions that may optimize the outcomes of VR interventions includes an adaptive training mechanism to maximize engagement and sustain motivation, more realistic portrayal of scenarios by enhancing both software and hardware technologies to increase emotional engagement, and developing VR encounters which align to the likely activities, environment, past experiences and learning needs of its end-users. A range of populations including caregivers, healthcare staff and vocations involving public fronting roles such as those in public transport can benefit from effective VR interventions in this area of training. VR holds promise for training of de-escalation of aggression but further discretion should be exercised in prioritizing the areas that would benefit most from VR interventions. More randomized controlled studies are required in specific population groups, for example patients taking into account their demographics and relevant factors such as comorbidities in order to enable effective implementation of such VR interventions. In addition, further cost-effectiveness studies will be important to evaluate the feasibility of implementation of VR interventions in the public and healthcare domains.

## Data Availability

The original contributions presented in the study are included in the article/Supplementary material, further inquiries can be directed to the corresponding author.
