# Supplementary material for: The utilization of virtual reality in the training of de-escalation of aggression for both providers and users of public and healthcare services from the new millennium to the COVID-19 era: a systematic review
Source: Front Med (Lausanne). 2026 Feb 5;13:1657986. doi: 10.3389/fmed.2026.1657986 (PMC12917911; doi:10.3389/fmed.2026.1657986)
Supplement: Supplementary file 1 [file Supplementary_file_1.pdf]

Quality appraisal for Randomised Controlled Trials

| No | Author | Title | Q1 | Q2 | Q3 | Q4 | Q5 | Q6 | Q7 | Q8 | Q9 | Q10 | Q11 | Q12 | Q13 | Total score |
|---|---|---|---|---|---|---|---|---|---|---|---|---|---|---|---|---|
| 1 | Smeijers et al.(2021) | Testing the Effects of a Virtual Reality Game for Aggressive Impulse Management | 2 | 2 | 2 | 2 | 2 | 0 | 2 | 2 | 2 | 2 | 2 | 2 | 0 | 22 |
| 2 | Tuente et al.(2020) | Virtual Reality Aggression Prevention Therapy (VRAPT) versus Waiting List Control for Forensic Psychiatric Inpatients: A Multicentre Randomised Controlled Trial | 2 | 2 | 2 | 0 | 1 | 0 | 1 | 1 | 2 | 2 | 2 | 1 | 0 | 16 |

Q1. Did the study address a clearly focused research question?
Q2. Was the assignment of participants to interventions randomised?
Q3. Were all participants who entered the study accounted for at its conclusion?
Q4. Were the participants ‘blind’ to intervention they were given?
Q5. Were the investigator ‘blind’ to intervention they were giving?
Q6. Were the people assessing/analysing outcome/s ‘blinded?
Q7. Were the study groups similar at the start of the randomised controlled trial?
Q8. Apart from the experimental intervention, did each study group receive the same level of care (that is, were they treated equally)?
Q9. Were the effects of intervention reported comprehensively?
Q10. Was the precision of the estimate of the intervention or treatment effect reported?
Q11. Do the benefits of the experimental intervention outweigh the harms and costs?
Q12. Can the results be applied to your local population/in your context?
Q13. Would the experimental intervention provide greater value to the people in your care than any of the existing interventions?

Quality appraisal for cohort studies

| No | Author | Title | Q1 | Q2 | Q3 | Q4 | Q5 | Q6 | Q7 | Q8 | Q9 | Q10 | Q11 | Q12 | Q13 | Total |
|---|---|---|---|---|---|---|---|---|---|---|---|---|---|---|---|---|
| 1 | Alsem et al.(2021) | Using Virtual Reality to Treat Aggressive Behaviour Problems in Children: a Feasibility Study | 2 | 1 | 2 | 2 | 1 | 1 | 1 | 2 | 1 | 1 | 1 | 1 | 2 | 18 |
| 2 | Halem et al.(2019) | Virtual Reality Training for Professionals to Support Prevention of Patient Aggression Incidents in the Workplace: Preliminary Results in Mental Healthcare Employees | 2 | 1 | 1 | 1 | 1 | 1 | 1 | 1 | 1 | 1 | 1 | 1 | 2 | 15 |
| 3 | Ryu et al.(2016) | Effects of an Anger Management Virtual Reality Cognitive Behavioural Therapy Program on EEG Patterns Among Destructive and Impulse-Control Diosrder Patients | 2 | 2 | 2 | 1 | 1 | 0 | 1 | 1 | 1 | 1 | 1 | 2 | 1 | 17 |
| 4 | Bosse et al.(2016) | An Intelligent System for Aggression De-escalation Training | 2 | 1 | 2 | 2 | 0 | 0 | 1 | 2 | 0 | 1 | 1 | 1 | 1 | 14 |

| | | | | | | | | | | | | | | | | |
|---|---|---|---|---|---|---|---|---|---|---|---|---|---|---|---|---|
| 5 | Bosse et al. (2015) | Evaluation of a Virtual Training Environment for Aggression De-escalation | 2 | 2 | 2 | 2 | 0 | 0 | 2 | 1 | 1 | 1 | 1 | 1 | 1 | 16 |
| 6 | Bosse et al.(2015) | The Effect of Simulated Threat on Task Performance During Emotion Recognition | 2 | 1 | 2 | 2 | 1 | 1 | 1 | 2 | 1 | 1 | 1 | 2 | 1 | 18 |

Q1. Did the study address a clearly focused issue?
Q2. Was the cohort recruited in an acceptable way?
Q3. Was the exposure accurately measured to minimise bias?
Q4. Was the outcome accurately measured to minimise bias?
Q5. Have the authors identified all important confounding factors?
Q6. Have they taken account of the confounding factors in the design and/or analysis?
Q7. Was the follow up of subjects complete enough?
Q8. Was the follow up of subjects long enough?
Q9. How precise are the results?
Q10. Do you believe the results?
Q11. Can the results be applied to the local population?
Q12. Do the results of this study fit with other available evidence?
Q13. What are the implications of this study for practice?

Quality appraisal for qualitative studies

| No | **Author** | **Title** | **Q1** | **Q2** | **Q3** | **Q4** | **Q5** | **Q6** | **Q7** | **Q8** | **Q9** | **Q10** | **Total score** |
|---|---|---|---|---|---|---|---|---|---|---|---|---|---|
| 1 | Garcia et al.(2021) | Exploring Perceptions of Bystander Intervention Training using Virtual Reality | 2 | 2 | 2 | 1 | 2 | 1 | 2 | 1 | 2 | 1 | 16 |
| 2 | Kim (2021) | Development and Effect of Virtual Reality Practice Program for Improving Practical Competency of Caregivers Specialising in Dementia | 2 | 2 | 2 | 2 | 2 | 0 | 2 | 1 | 2 | 1 | 16 |
| 3 | Blankendaal et al. (2018) | A Virtual Reality Application for Aggression De-escalation Training in Public Transport | 2 | 2 | 1 | 1 | 1 | 0 | 0 | 1 | 2 | 1 | 11 |
| 4 | Blankendaal et al.(2018) | Using Run-Time Biofeedback During Virtual Agent-Based Aggression De-escalation Training | 2 | 2 | 2 | 1 | 1 | 1 | 0 | 1 | 2 | 2 | 14 |

Q1. Was there a clear statement of the aims of the research?
Q2. Is a qualitative methodology appropriate?
Q3. Was the research design appropriate to address the aims of the research?
Q4. Was the recruitment strategy appropriate to the aims of the research?
Q5. Was the data collected in a way that addressed the research issue?
Q6. Has the relationship between researcher and participants been adequately considered?
Q7. Have ethical issues been taken into consideration?
Q8. Was the data analysis sufficiently rigorous?
Q9. Is there a clear statement of findings?
Q10. How valuable is the research?
